# Collaborating to Compete: Blood Profiling Atlas in Cancer (BloodPAC) Consortium

**DOI:** 10.1002/cpt.666

**Published:** 2017-04-12

**Authors:** RL Grossman, B Abel, S Angiuoli, JC Barrett, D Bassett, K Bramlett, GM Blumenthal, A Carlsson, R Cortese, J DiGiovanna, B Davis‐Dusenbery, R Dittamore, DA Eberhard, P Febbo, M Fitzsimons, Z Flamig, J Godsey, J Goswami, A Gruen, F Ortuño, J Han, D Hayes, J Hicks, D Holloway, D Hovelson, J Johnson, H Juhl, R Kalamegham, R Kamal, Q Kang, GJ Kelloff, M Klozenbuecher, A Kolatkar, P Kuhn, K Langone, R Leary, P Loverso, H Manmathan, A‐M Martin, J Martini, D Miller, M Mitchell, T Morgan, R Mulpuri, T Nguyen, G Otto, A Pathak, E Peters, R Philip, E Posadas, D Reese, MG Reese, D Robinson, A Dei Rossi, H Sakul, J Schageman, S Singh, HI Scher, K Schmitt, A Silvestro, J Simmons, T Simmons, J Sislow, A Talasaz, P Tang, M Tewari, S Tomlins, H Toukhy, HR Tseng, M Tuck, A Tzou, J Vinson, Y Wang, W Wells, A Welsh, J Wilbanks, J Wolf, L Young, JSH Lee, LC Leiman

**Affiliations:** ^1^ Center for Data Intensive Science University of Chicago Chicago Illinois USA; ^2^ Genomic Health Redwood City California USA; ^3^ Personal Genome Diagnostics Baltimore Maryland USA; ^4^ AstraZeneca Waltham Massachusetts USA; ^5^ Celgene Seattle Washington USA; ^6^ Thermo Fisher Scientific Austin Texas USA; ^7^ Center for Drug Evaluation and Research Food and Drug Administration Silver Springs Maryland USA; ^8^ Department of Molecular and Medical Pharmacology Crump Institute for Molecular Imaging, University of California Los Angeles California USA; ^9^ Seven Bridges Cambridge Massachusetts USA; ^10^ Epic Research and Diagnostics San Diego California USA; ^11^ University of Michigan Ann Arbor Michigan USA; ^12^ Indivumed GmbH Hamburg Germany; ^13^ Genentech Washington District of Columbia USA; ^14^ Omicia Oakland California USA; ^15^ Office of the Director National Cancer Institute Bethesda Maryland USA; ^16^ Novartis Institute for Biomedical Research Cambridge Massachusetts USA; ^17^ Novartis Pharmaceuticals East Hanover New Jersey USA; ^18^ Memorial Sloan Kettering Cancer Center New York New York USA; ^19^ Provista Diagnostics Inc. New York New York USA; ^20^ Foundation Medicine Cambridge Massachusetts USA; ^21^ Center for Device and Radiological Health Food and Drug Administration Silver Springs Maryland USA; ^22^ CytoLumina, Inc. Los Angeles California USA; ^23^ Pfizer San Diego California USA; ^24^ Guardant Health, Inc. Redwood City California USA; ^25^ Open Commons Consortium Chicago Illinois USA; ^26^ Sage Bionetworks Seattle Washington USA; ^27^ BloodPAC Chicago Illinois USA; ^28^ Cedar‐Sinai Medical Center Los Angeles California USA; ^29^ Crump Institute for Molecular Imaging University of California Los Angeles California USA; ^30^ Thermo Fisher Scientific Waltham Massachusetts USA; ^31^ Thermo Fisher Scientific Carlsbad California USA; ^32^ Genentech South San Francisco California USA

## Abstract

The cancer community understands the value of blood profiling measurements in assessing and monitoring cancer. We describe an effort among academic, government, biotechnology, diagnostic, and pharmaceutical companies called the Blood Profiling Atlas in Cancer (BloodPAC) Project. BloodPAC will aggregate, make freely available, and harmonize for further analyses, raw datasets, relevant associated clinical data (e.g., clinical diagnosis, treatment history, and outcomes), and sample preparation and handling protocols to accelerate the development of blood profiling assays.

The main goal of Precision Medicine (PM) is the customization of healthcare, with medical decisions, practices, and/or products being tailored to an individual patient. Several national efforts, such as the Precision Medicine Initiative (PMI),^1^ are working to integrate PM into biomedical research, drug and diagnostic test development, and patient care to achieve the highest levels of accuracy and precision in the treatment of a patient. Multiple PM stakeholders, including patients, physicians, healthcare administrators, regulators, and payers are hoping for: 1) breakthrough therapies; 2) breakthrough diagnostics; and 3) greater value to patients and the healthcare system by achieving superior clinical outcomes through identification of patients most likely to benefit, thereby sparing the drug toxicities and high cost associated with ineffective treatments. Thus far, oncology has been one of the biggest beneficiaries of PM, where cancers once perniciously progressive have been significantly slowed, and even cured, through the use of evolving technologies that enable the matching of therapies to an individual's tumor biology.

Thanks to these significant scientific advances, we know that tumors shed cells and a variety of chemical signals into the bloodstream, leaving behind small hints that can identify the cancer phenotype and genotype, the organ of origin, and the evolutionary state of cancer as a result of its natural progression and/or treatment pressures. For this reason, researchers are especially interested in developing new technologies to use this knowledge to transform how we detect and diagnose cancer, and how we predict and monitor response to therapeutic intervention. This provides the foundation for a future where simple blood draws could help physicians and patients more accurately and successfully detect and manage the disease. This approach, commonly described as liquid biopsies, provides a less invasive, more easily replicable, and potentially more informative alternative to standard tissue biopsies. Liquid biopsies are experienced by the patient as a simple blood test as compared to an often painful bone biopsy or more invasive open biopsy under anesthesia.

The value of blood profiling measurements in assessing and monitoring cancer patient status includes: repeated access to tumor material when tissue biopsies are impractical, more comprehensive assessment of tumor biology compared to sampling of one specific tumor locus, and more practical assessment of the molecular evolution of cancer throughout a patient's treatment. There has been significant academic and commercial activity in the development of liquid biopsies that are both cell‐based, such as circulating tumor cells (CTC), and cell‐free, such as circulating tumor DNA (ctDNA), isolation and analysis platforms, as well as technologies to assay for exosomes and other extracellular vesicles.

At this time, two liquid biopsies have been clinically validated and approved by the US Food and Drug Administration (FDA) as a companion diagnostic. Both test for mutations in the *EGFR* gene for patients with metastatic non‐small cell lung cancer (NSCLC). The cobas *EGFR* Mutation Test v2 (Roche Molecular Systems, Nutley, NJ) tests for exon 19 deletions or exon 21 (L858R) substitution mutations in the *EGFR* gene to identify patients eligible for treatment with Tarceva (erlotinib).^2^ The same test also can detect the T790M mutation in the *EGFR* gene to identify patients for treatment with Tagrisso (osimertinib).^3^

Clinicians are currently restricted in their ability to include liquid biopsy analysis as a part of clinical care (e.g., for serial monitoring), other than for ctDNA single draws, due to lack of sufficient evidence for payer reimbursement. There is growing concern that ctDNA analysis may follow the same path as first‐generation CTC methods^4^ because the data needed to demonstrate the level of evidence that the FDA and payers believe is needed for clinical efficacy currently exists for only the two indications just mentioned. While many blood profiling platforms exist for research use only (RUO), questions remain regarding the performance characteristics and clinical validation of these platforms, and standard protocols for sample collection, processing, and analysis remain to be established. This information is a prerequisite to the design of clinical studies to demonstrate clinical utility.^5^ Akin to the concept used by The Cancer Genome Atlas (TCGA) launched in 2006, this foundational information can be developed through collaboration across stakeholders and sharing of very early‐stage research. Establishing a comprehensive knowledge base in the liquid biopsy space will provide an invaluable reference for anyone entering the field, help develop the evidence required to support clinical utility, assay reproducibility, and provide support for FDA approval and payer reimbursement.

Following the advent of the Cancer Moonshot Initiative,^6^ the Blood Profiling Atlas in Cancer (BloodPAC, http://bloodpac.org) Consortium was launched on October 17, 2016 to aggregate, make freely available, and harmonize data for further analysis: 1) data from CTC, ctDNA, proteins including tumor associated autoantibodies, and exosome assays; 2) associated clinical data, such as clinical diagnosis, treatment history and outcomes; and 3) sample collection, preparation, and handling protocols. This collaborative effort brings together representatives from academia, private foundations, industry, and the government to accelerate the exploration, implementation, and assessment of potential clinical utility of liquid biopsies with the aim to understand the temporal evolution of a patient's disease. Currently, 25 organizations are participating in the Consortium, with additional members joining in 2017.

The initial project goals were threefold. First, participants have pledged and started to contribute data that underpin the preanalytical and analytical criteria for different liquid biopsy test platforms. The second goal is to develop a prototype BloodPAC Data Commons and analysis cloud, which will be based in part on the open source software developed for the NCI Genomic Data Commons and Cancer Genomics Cloud Pilots.^7^ Data commons and analysis clouds colocate the data, storage, and computing infrastructure with commonly used tools, applications and services to create interoperable resources for the research and clinical communities.^7^ With a data commons and analysis cloud, researchers can collaboratively analyze data in a scalable and reproducible manner using existing or novel tools, without first downloading it and setting up their own analysis environments.^7^ The BloodPAC Data Commons shares a data model with the NCI Genomic Data Commons and employs the same core web services, such as those providing unique digital IDs for data, so that linking the two commons and building applications that access data from both is straightforward. The third goal is for the BloodPAC team to summarize the information from the commons to provide a “how to” for the scientific/medical community on the minimal criteria required for establishing liquid biopsy testing in collaboration with the FDA and the College of American Pathologists.

As a proof of concept, three working groups of BloodPAC coordinated the initial deposition of preanalytical datasets and sample preparation protocols. The Data Working Group, co‐chaired by Robert Grossman and Brandi Davis‐Dusenbery, developed a secure and compliant data commons (the BloodPAC Data Commons) to store, analyze, and share the datasets, an initial data model (**Figure** 1) for storing, accessing, and querying the commons, data use agreements that were completed by 13 of 23 stakeholders during the first 60 days of the project, and a preliminary version of a pipeline for analyzing some of the submitted data. The Technology Applications Working Group, co‐chaired by Peter Kuhn, Muneesh Tewari, and John Simmons, is working alongside the Samples Working Group, which is co‐chaired by Howard Scher, Craig Shriver, and Anne‐Marie Martin, to review the analytical and preliminary clinical assay validation methodology for data to be submitted to the BloodPAC Data Commons. Collectively, this first set of data will drive important analysis on preanalytical assessments, including questions about the specimen before it is analyzed, such as specimen collection, handling, processing, storage, and annotation. This will provide a solid foundation upon which future BloodPAC work will build.

**Figure 1:**
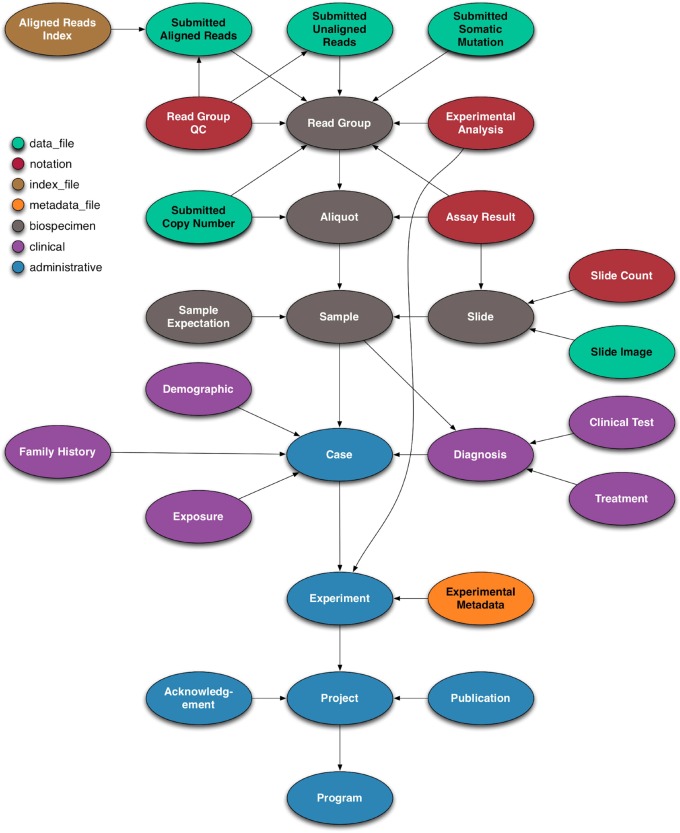
BloodPAC Data Model.

The BloodPAC Consortium is a unique opportunity for the public, researchers, and industry to all gain by collaboration. The primary beneficiaries are patients who will gain earlier access to blood‐based tests to detect and characterize cancer. Industry has some of the most to offer, and gain, from BloodPAC. Pharmaceutical, diagnostic, and tool companies all have datasets to share. Analysis companies have algorithms and expertise to share. However, prior to the BloodPAC, these groups had never been in the same room, working on the same problems, together with government regulators and academic researchers. Researchers also stand to immediately gain from the BloodPAC. One of the most intractable problems of modern science is the disincentive to publish negative results, causing researchers around the world to repeat the mistakes of their colleagues. The BloodPAC Consortium is sharing very early‐stage research information about how to best collect and analyze these specimens, and, critically, it is also sharing what has not worked.

Moving forward, BloodPAC has as its mandate to accelerate the development and approval of liquid biopsy assays to improve the outcomes of patients with cancer. To do so, the BloodPAC Consortium will develop and operate a collaborative infrastructure that enables sharing of information between stakeholders in industry, academia, and regulatory agencies and to conduct clinical validation studies designed to improve the outcomes for patients with cancer. The ultimate goal from this project will be to create a “patient disease and treatment path” so that the scientific and clinical communities have a better understanding of how a disease progresses and how to optimize the choice of therapy at particular points in time. Thus, the BloodPAC Consortium could bring value to all precision medicine stakeholders because: 1) patients may have access to easier, minimally invasive, disease monitoring tools to inform the best treatment decisions; 2) physicians may be able to monitor patients more frequently and make more informed decisions more rapidly; 3) assay developers will have access to information that will reduce their need to reproduce data that already exists and to enable a focus on the generation of new knowledge; 4) the summary of assay performance elements could provide a foundation for liquid biopsy validation and qualification requirements to meet regulatory approval; and 5) paths for reimbursement of testing and treating will be more apparent for payers. Precision Medicine requires the close collaboration between a complex system of stakeholders, each of whom applies their expertise to better understand and address the varied needs of patients. Blood PAC is uniquely positioned through engagement of all stakeholders to be successful in bringing liquid biopsies into broad clinical care.

## ACKNOWLEDGMENTS

We thank C. Compton, B. Conley, J. Doroshow, T. Lively, D. Lowy, H. Rodriguez, A. Schade, C. Shriver, S. Somiari, and M. Williams for their participation in ongoing discussions with BloodPAC.

## CONFLICT OF INTEREST

The authors declare no conflicts of interest.

## References

[1] White House (US) . Remarks by the President in State of the Union Address [Internet]. Washington, DC: White House (US); [cited 06 February 2016]. Available from: <https://obamawhitehouse.archives.gov/the‐press‐office/2015/01/20/remarks‐president‐state‐union‐address‐january‐20‐2015> (2015).

[2] cobas EGFR Mutation Test v2, June 2, 2016 [cited 06 February 2016]. Available from: <http://www.fda.gov/Drugs/InformationOnDrugs/ApprovedDrugs/ucm504540.htm>.

[3] List of Cleared or Approved Companion Diagnostic Devices (In Vitro and Imaging Tools) [cited 06 February 2016]. Available from: <http://www.fda.gov/MedicalDevices/ProductsandMedicalProcedures/InVitroDiagnostics/ucm301431.htm>.

[4] Parkinson, D.R. *et al* Considerations in the development of circulating tumor cell technology for clinical use. J. Transl. Med. 10, 138–157 (2012). 22747748 10.1186/1479-5876-10-138 PMC3478228

[5] Parkinson, D.R. *et al* Evidence of clinical utility: an unmet need in molecular diagnostics for patients with cancer. Clin. Cancer Res. 20, 1428–1444 (2014). 24634466 10.1158/1078-0432.CCR-13-2961

[6] White House (US) . Remarks of President Barack Obama — State of the Union Address As Delivered [Internet]. Washington, DC: White House (US); [cited 06 February 2016]. Available from: <https://obamawhitehouse.archives.gov/the‐press‐office/2016/01/12/remarks‐president‐barack‐obama‐–‐prepared‐delivery‐state‐union‐address> (2016).

[7] Grossman, R.L. *et al* Toward a shared vision for cancer genomic data. N. Engl. J. Med. 375, 1109–1112 (2016). 27653561 10.1056/NEJMp1607591 PMC6309165

